# Regulation of Expression of Autophagy Genes by Atg8a-Interacting Partners Sequoia, YL-1, and Sir2 in *Drosophila*

**DOI:** 10.1016/j.celrep.2020.107695

**Published:** 2020-05-26

**Authors:** Anne-Claire Jacomin, Stavroula Petridi, Marisa Di Monaco, Zambarlal Bhujabal, Ashish Jain, Nitha C. Mulakkal, Anthimi Palara, Emma L. Powell, Bonita Chung, Cleidiane Zampronio, Alexandra Jones, Alexander Cameron, Terje Johansen, Ioannis P. Nezis

**Affiliations:** 1 School of Life Sciences, University of Warwick, CV4 7AL Coventry, UK; 2 Molecular Cancer Research Group, Institute of Medical Biology, University of Tromsø–The Arctic University of Norway, 9037 Tromsø, Norway; 3 Department of Molecular Cell Biology, Institute for Cancer Research, Oslo University Hospital, Montebello, 0379 Oslo, Norway; 4 Centre for Cancer Cell Reprogramming, Institute of Clinical Medicine, Faculty of Medicine, University of Oslo, Montebello, 0379 Oslo, Norway

**Keywords:** acetylation, autophagy, LIR motif, nucleus, transcription, LC3/Atg8

## Abstract

Autophagy is the degradation of cytoplasmic material through the lysosomal pathway. One of the most studied autophagy-related proteins is LC3. Despite growing evidence that LC3 is enriched in the nucleus, its nuclear role is poorly understood. Here, we show that *Drosophila* Atg8a protein, homologous to mammalian LC3, interacts with the transcription factor Sequoia in a LIR motif-dependent manner. We show that Sequoia depletion induces autophagy in nutrient-rich conditions through the enhanced expression of autophagy genes. We show that Atg8a interacts with YL-1, a component of a nuclear acetyltransferase complex, and that it is acetylated in nutrient-rich conditions. We also show that Atg8a interacts with the deacetylase Sir2, which deacetylates Atg8a during starvation to activate autophagy. Our results suggest a mechanism of regulation of the expression of autophagy genes by Atg8a, which is linked to its acetylation status and its interaction with Sequoia, YL-1, and Sir2.

## Highlights

• Transcription factor Sequoia is a negative regulator of autophagy
• Sequoia interacts with Atg8a via a LIR motif
• Atg8a interacts with YL-1, a subunit of a nuclear acetyltransferase complex
• Sir2 interacts with and deacetylates Atg8a during starvation

## Graphical Abstract

**Figure:**
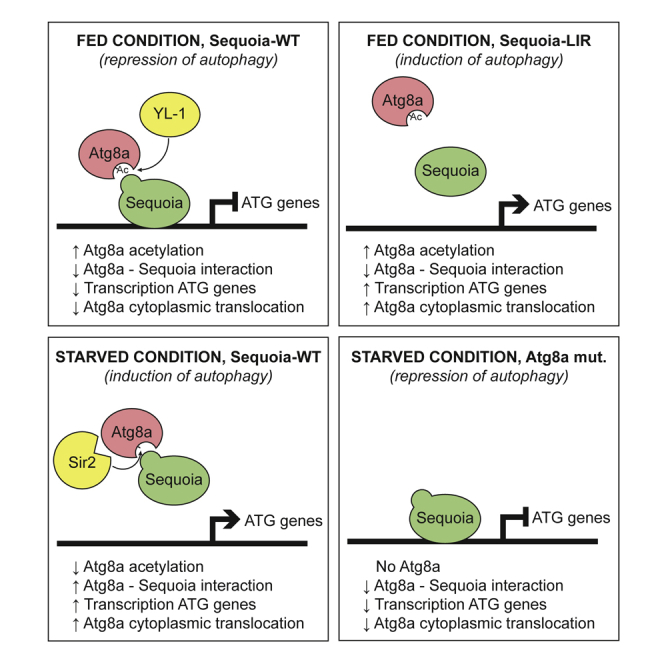

## Teaser

Jacomin et al. show that Atg8a interacts with transcription factor Sequoia. Depletion of Sequoia induces autophagy through enhanced expression of autophagy genes. Atg8a also interacts with YL-1, a component of a nuclear acetyltransferase complex, and deacetylase Sir2. Sir2 deacetylates Atg8a during starvation to activate autophagy.

## Introduction

Autophagy is a fundamental, evolutionary conserved process in which cytoplasmic material is degraded through the lysosomal pathway. It is a cellular response during nutrient starvation; yet, it is also responsible in basal conditions for the removal of aggregated proteins and damaged organelles and therefore plays an important role in the maintenance of cellular homeostasis (Ambrosio et al., 2019, Dikic and Elazar, 2018, Füllgrabe et al., 2014, Gatica et al., 2018, Lamb et al., 2013, Sakamaki et al., 2017, Sakamaki et al., 2018). There are three main types of autophagy: macroautophagy, microautophagy, and chaperone-mediated autophagy (Dikic and Elazar, 2018, Gatica et al., 2018, Lamb et al., 2013). Macroautophagy, referred to as autophagy, is the best-described type of autophagy. During macroautophagy, cytoplasmic material is isolated into double-membrane vesicles called autophagosomes. Autophagosomes eventually fuse with lysosomes, allowing for the degradation of cargoes by lysosomal hydrolases. The products of degradation are transported back into the cytoplasm through lysosomal membrane permeases and can be reused by the cell (Dikic and Elazar, 2018, Gatica et al., 2018, Lamb et al., 2013).

One of the most important and well-studied autophagy-related proteins is LC3 (microtubule-associated protein 1 light chain 3, called Atg8 in yeast and *Drosophila*), which participates in autophagosome formation. LC3 interacts with LIR (LC3-interacting region) motifs also known as AIM (Atg8-interacting motifs) on selective autophagy receptors that carry cargo for degradation, and is one of the most widely used markers of autophagy (Gatica et al., 2018, Kabeya et al., 2000). Despite growing evidence that LC3 is enriched in the nucleus, little is known about the mechanisms involved in targeting LC3 to the nucleus and the nuclear components with which it interacts (Dou et al., 2015, Guo et al., 2018, Huang et al., 2015, Kabeya et al., 2000, Klionsky et al., 2016, Kraft et al., 2016).

Here, we show that *Drosophila* Atg8a protein, homologous to mammalian LC3 and yeast Atg8, interacts with the transcription factor Sequoia in a LIR motif-dependent manner that is not responsible for the degradation of Sequoia. We show that Sequoia depletion induces autophagy in nutrient-rich conditions through the enhanced expression of autophagy genes. We also found that Atg8a is acetylated and interacts with YL-1, a component of the NuA4/Tip60 nuclear acetyltransferase complex. We show that Atg8a interacts with the deacetylase Sir2, which deacetylates Atg8a during starvation to activate autophagy. Our results suggest a novel mechanism of regulation of autophagy gene expression by Atg8a, which is linked to its acetylation status and its interaction with Sequoia, YL-1, and Sir2.

## Results

### Transcription Factor Sequoia Is an Atg8a-Interacting Protein

**Figure 1:**
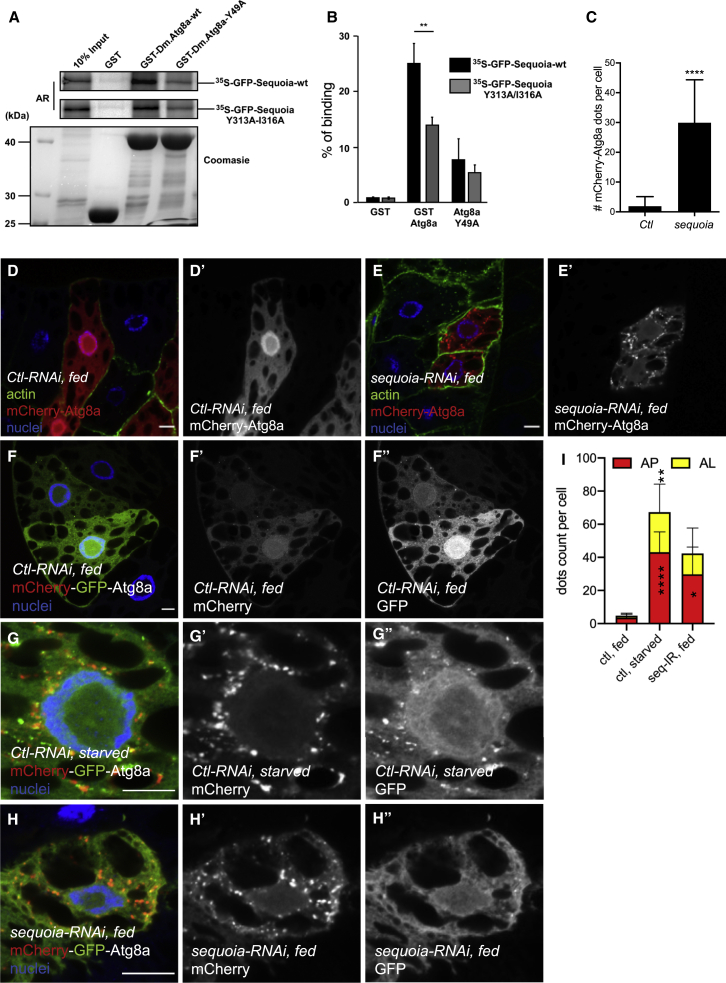
Sequoia Binds to Atg8a via a LIR Motif and Negatively Regulates Autophagy (A and B) Sequoia interacts with Atg8a in a LIR motif-dependent manner. (A) GST-pull-down assay between GST-tagged Atg8a-WT or Atg8a-LDS mutant (Y49A), and radiolabeled GFP-Sequoia-WT or GFP-Sequoia-LIR mutant (Y313A/316A). GST was used as negative control. Quantification of the binding is shown in (B). Statistical significance was determined using Student’s t test; ^∗∗^p < 0.01. (C–E) Confocal sections of larval fat bodies clonally expressing the autophagy marker mCherry-Atg8a (red) in combination with a control RNAi (D) or a *sequoia* RNAi (E). Fixed fat bodies where stained for cortical actin (green) and nuclei (blue). Scale bar: 10 μm. (C) Quantification of the number of mCherry-Atg8a dots per cell. Bars denote means ± SDs. Statistical significance was determined using Student’s t test; ^∗∗∗∗^p < 0.0001. (F–I) Confocal sections of larval fat bodies clonally expressing the autophagy flux marker GFP-mCherry-Atg8a (red and green) in combination with a control-RNAi in fed (F) or starved (G) or *sequoia*-RNAi in fed larvae. Sequoia depletion induces accumulation of autolysosomes. (H). Fixed fat bodies where stained for nuclei (blue). Scale bar: 10 μm. Quantification of the yellow (AP, autophagosome) and red only (AL, autolysosomes) puncta per cell. Statistical significance was determined using 1-way ANOVA test; ^∗^p < 0.05, ^∗∗^p < 0.01, ^∗∗∗∗^p < 0.001. Genotypes for D: yw hs-Flp;Ac > CD2 > GAL4/+;UAS-mCherry-Atg8a/UAS-luc-RNAi. (E) yw hs-Flp;Ac > CD2 > GAL4/+; UAS-mCherry-Atg8a/UAS-sequoia-RNAi. (F and G) ctl: Cg-GAL4/+;UAS-luc-RNAi/+, seq-RNAi: Cg-GAL4/+; UAS-sequoia-RNAi/+. (H) yw hs-Flp;Ac > CD2 > GAL4/+;UAS-mCherry-GFP-Atg8a/UAS-sequoia-RNAi.

To identify novel Atg8a-interacting proteins in *Drosophila*, we screened the *Drosophila* proteome for LIR motif-containing proteins using the iLIR software that we developed (Jacomin et al., 2016, Kalvari et al., 2014). We found that the transcription factor Sequoia (CG32904) has a predicted LIR motif at position 311–316 with the sequence EEYQVI (Figure S1A) (Jacomin et al., 2016, Kalvari et al., 2014, Popelka and Klionsky, 2015). Sequoia contains two zinc-finger domains that are homologous to the DNA-binding domain of Tramtrack and has been shown to regulate neuronal morphogenesis (Brenman et al., 2001). We confirmed the direct interaction between Sequoia and Atg8a using glutathione S-transferase (GST)-pull-down binding assays (Figures 1A and 1B). This interaction was significantly reduced when we used a mutant of Atg8a in which the LIR motif docking site (LDS) (Y49A) was impaired, indicating that the interaction between Sequoia and Atg8a is LIR motif dependent (Birgisdottir et al., 2013, Ichimura et al., 2008, Jain et al., 2015). Furthermore, point mutations of the Sequoia LIR motif in positions 313 and 316 by alanine substitutions of the aromatic and hydrophobic residues (Y313A and I316A) reduced its binding to Atg8a (Figures 1A and 1B). Using GST-pull-down assays, we also observed that the mammalian homolog of Sequoia, KDM4A, interacts with GABARAP and GABARAP-L1 (the closest mammalian homologs to Atg8a), suggesting evolutionary conservation of the interaction (Figures S1B and S1C). However, mutation of the putative LIR motifs of KDM4A did not abrogate its interaction with GABARAP-L1 (Figure S1C).

Given the observed interaction between Sequoia and Atg8a, we examined whether Sequoia is degraded by autophagy. Western blot analysis showed that endogenous Sequoia is not accumulated in *Atg8a* and *Atg7* mutants compared to wild-type (WT) flies (Figure S1D). These results indicate that Sequoia is an Atg8a-interacting protein and that this interaction is LIR motif dependent. In spite of its interaction with Atg8a, Sequoia is not a substrate for autophagic degradation.

### Sequoia Is a Negative Transcriptional Regulator of Autophagy

To examine the role of Sequoia in autophagy, we silenced *sequoia* using RNAi alongside the expression of the autophagic marker mCherry-Atg8a (Chang and Neufeld, 2009, Nezis et al., 2009). We observed a significant increase in the number of mCherry-Atg8a puncta in Sequoia-depleted fat body cells in fed conditions compared to control cells (Figures 1C–1E). An accumulation of Atg8a^+^ puncta in the cell can result either from an induction or a blockade of the autophagic flux. To make the distinction between these two possibilities, we made use of a tandem-tagged Atg8a (GFP-mCherry-Atg8a) (Nezis et al., 2010). In cells expressing RNAi against *sequoia*, we noticed an increased accumulation of red puncta that lack GFP fluorescence compared to the control, suggesting an induction of autophagic flux (Figures 1F–1I). To further test the activation of autophagy in Sequoia-depleted cells, we used western blotting and examined the presence of lipidated Atg8a (Atg8a-II). We observed that Atg8a-II accumulates more in Sequoia-depleted larvae compared to controls (Figures S2A and S2B).

**Figure 2:**
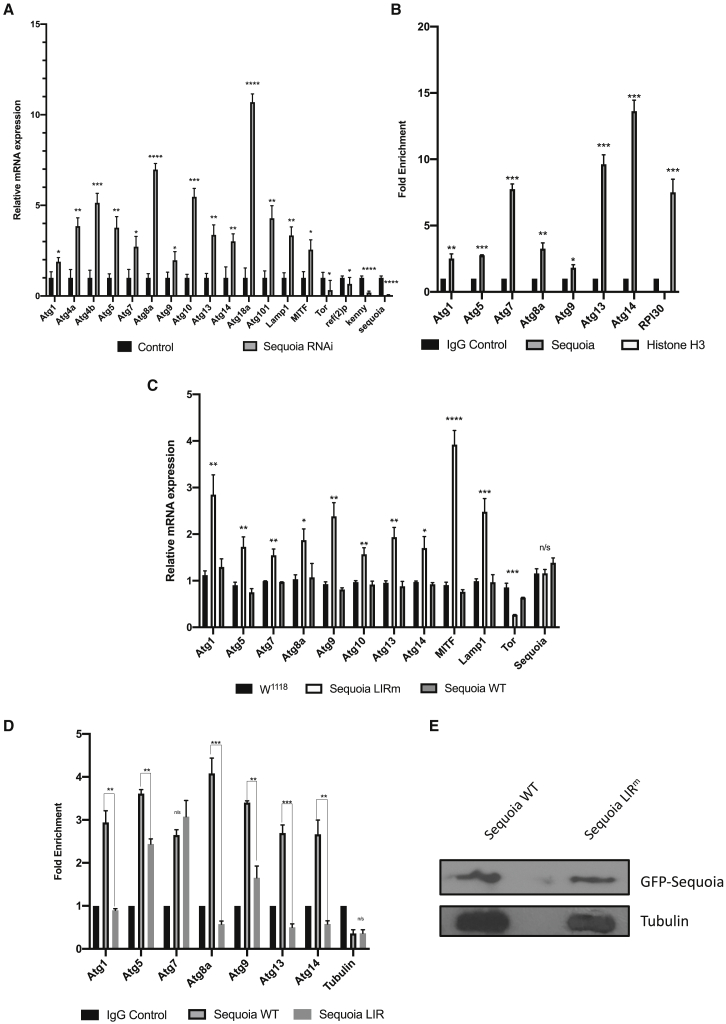
Sequoia Negatively Regulates Autophagy Genes (A) Analysis of the mRNA levels of autophagy-associated genes, sequoia, and autophagy receptors (Kenny and Ref(2)P) in control (luciferase RNAi) and Sequoia-depleted fat bodies in fed conditions, using real-time qPCR. (B) Analyses of Sequoia binding to the promoter of autophagy genes in fed conditions, as detected by ChIP (chromatin immunoprecipitation) using a Sequoia antibody. ChIP DNA values were normalized to input DNA using the 2-ΔΔCt method. Fold enrichment values are shown relative to the immunoglobulin G (IgG) control. Histone H3 enrichment to *RPL30* was used as a positive control. (C) Analysis of the mRNA level of autophagy-associated genes in W^1118^, Sequoia LIR mutant, and Sequoia WT fat bodies in fed conditions, using real-time qPCR. (D) Analyses of Sequoia binding to the promoter of autophagy genes in fed conditions, as detected by ChIP using a GFP antibody. ChIP DNA values were normalized to input DNA using the 2-ΔΔCt method. Fold enrichment values are shown relative to the IgG control. Tubulin was used as a non-autophagy-related gene control. (E) Expression of GFP-Sequoia protein in Sequoia WT and Sequoia LIR mutant following heat shock. All data shown as means ± SDs, n = 3 independent experiments. Statistical significance was determined using Student’s t test; ^∗^p < 0.05 and ^∗∗∗^p < 0.005. Genotypes for A: ctl: Cg-GAL4/+;UAS-luc-RNAi/+, seq-RNAi: Cg-GAL4/+;UAS-sequoia-RNAi/+. (B): Cg-GAL4/+;W^1118^. (C—E) hs::Gal4/UAS-GFP-Sequoia-WT,hs::Gal4/UAS-GFP-Sequoia-LIRm.

We next examined whether the expression of autophagy genes was affected upon *Sequoia* knockdown. Real-time quantitative PCR (qPCR) analysis showed that the expression of numerous autophagy genes was increased when *sequoia* was silenced (Figure 2A). As Sequoia is a transcription factor and contains C2H2 zinc-finger domains involved in DNA binding, we performed a chromatin immunoprecipitation (ChIP) assay to test the ability of Sequoia to bind the promoter region of autophagy genes (promoter regions shown in Table S1). We found that Sequoia is enriched on the promoters of several autophagy genes, suggesting that Sequoia is acting as a repressor to negatively regulate autophagy (Figure 2B). These results show that Sequoia is a negative transcriptional regulator of autophagy.

### The Repressive Activity of Sequoia on Autophagy Depends on Its LIR Motif

**Figure 3:**
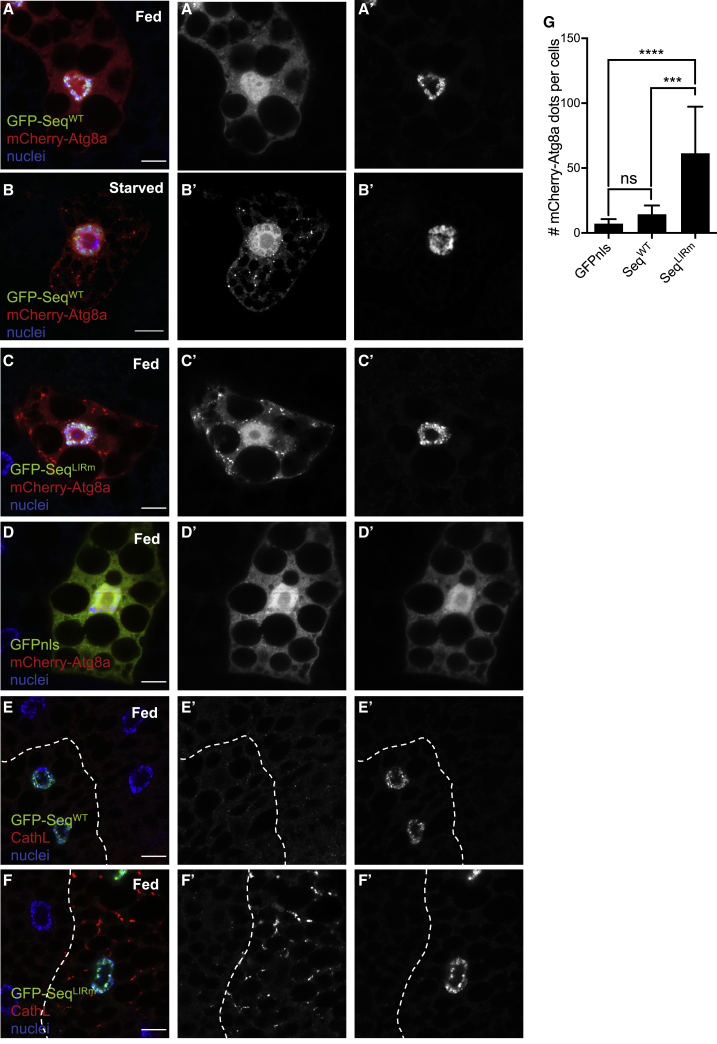
The LIR Motif of Sequoia Is Required to Repress Autophagy (A–D) Confocal sections of larval fat bodies clonally expressing the autophagy marker mCherry-Atg8a (red) in combination with GFP-Sequoia WT (A and B), GFP-Sequoia LIRm (C), or GFP-nls (D) (green). Larvae were well fed (A, C, and D) or starved for 4 h (B). Fixed fat bodies were stained for nuclei (blue). Scale bar: 10 μm. (E and F) Confocal sections of larval fat bodies clonally expressing GFP-Sequoia WT (E) and GFP-Sequoia LIRm (F) (green) and stained for cathepsin L (red). (G) Quantification of the number of mCherry-Atg8a dots per cell. Bars denote means ± SDs. Statistical significance was determined using 1-way ANOVA; ^∗∗∗^p < 0.001 and ^∗∗∗∗^p < 0.0001. Genotypes for A and B: yw hs-Flp;Ac > CD2 > GAL4/UAS-GFP-Sequoia-WT;UAS-mCherry-Atg8a/+. (C) yw hs-Flp;Ac > CD2 > GAL4/UAS-GFP-Sequoia-LIRm;UAS-mCherry-Atg8a/+. (D) yw hs-Flp;Ac > CD2 > GAL4/+;UAS-mCherry-Atg8a/UAS-GFPnls. (E) yw hs-Flp;Ac > CD2 > GAL4/UAS-GFP-Sequoia-WT. (F) yw hs-Flp;Ac > CD2 > GAL4/UAS-GFP-Sequoia-LIRm.

To evaluate whether the role of Sequoia in the negative transcriptional regulation of autophagy depends on its interaction with Atg8a via its LIR motif, we created transgenic flies allowing for the expression of GFP-tagged WT (GFP-Sequoia-WT) or LIR mutant (GFP-Sequoia-Y313A/I316A) Sequoia under the control of a UAS region. Expression of the Sequoia LIR mutant demonstrated the reduced presence of Sequoia on the promoter regions of autophagy genes and that correlates with a higher expression level of those genes (Figures 2C–2E). Both GFP-Sequoia-WT and GFP-Sequoia-Y313A/I316A proteins localized exclusively in the nucleus of fat body cells (Figures 3A and 3C). GFP-Sequoia-WT localized in the nucleus in both fed and starved conditions (Figures 3A and 3B). The expression of GFP-Sequoia-Y313A/I316A resulted in a significant increase in mCherry-Atg8a puncta in the cytoplasm in fed conditions, while the expression of WT Sequoia had no effect (Figures 3A, 3C, 3D, and 3G). In addition, mosaic analysis revealed that only cells expressing LIR-mutated Sequoia had an increase in the lysosomal marker cathepsin L (Figures 3E and 3F). These results suggest that Sequoia negatively regulates autophagy through its LIR motif-dependent interaction with Atg8a.

### Atg8a Interacts with YL-1, a Subunit of a Nuclear Acetyltransferase Complex, and Is Acetylated in Nutrient-Rich Conditions

**Figure 4:**
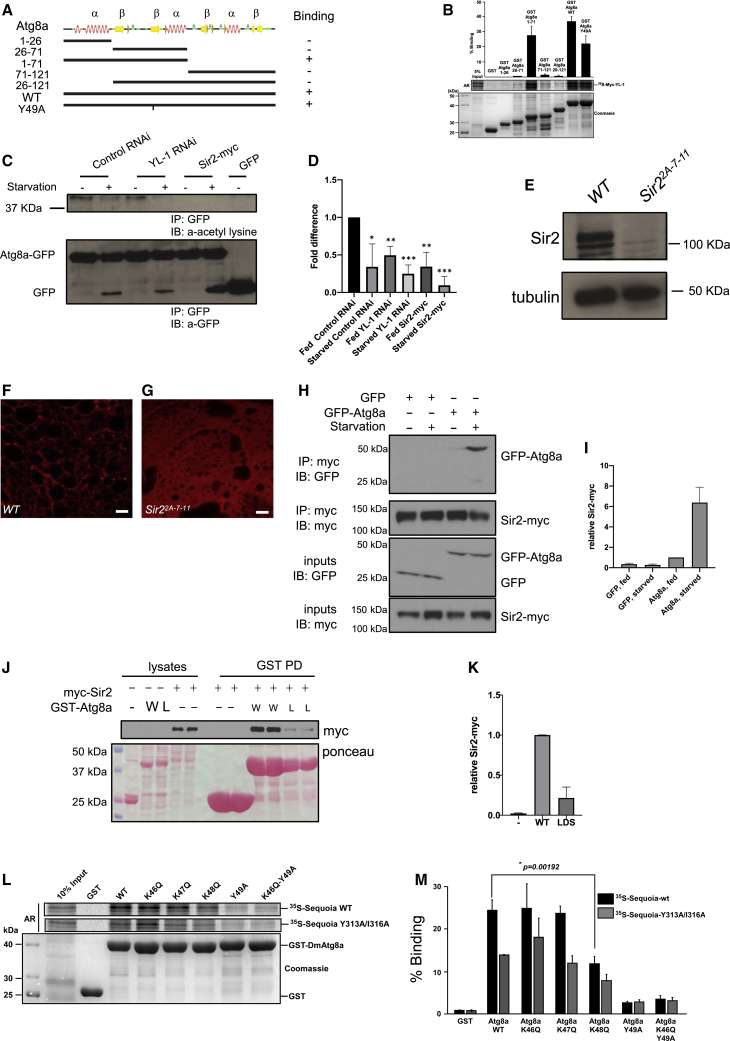
YL-1 and Sir2 Interact with Atg8a, Regulating Its Acetylation Status (A and B) YL-1 interacts with Atg8a in a non-LIR-dependent manner. (A) Overview of the secondary structure of Atg8a with deletion mutants and the Y49A LDS mutant of Atg8a used in GST-pull-down assays in (B). (B) GST-pull-down assays between GST-tagged WT Atg8a, Atg8a deletion mutants, and the LDS mutant (Y49A) and radiolabeled myc-YL-1. (C) Knockdown of the YL-1 acetyltransferase reduces the acetylation of Atg8a. Flies that were constitutively expressing GFP-Atg8a in their fat bodies were crossed with the control luciferase RNAi, the YL-1 RNAi line, and the Sir2-Myc line and their offspring were collected. Protein acetylation was determined by immunoprecipitation (IP) with a GFP antibody followed by western blotting (WB) for an antibody recognizing acetyl-lysine residues. IP was performed with 1 mg protein lysate from full larvae both in fed and starved (4 h in 20% sucrose) conditions. (D) Quantification of the quantity of Atg8a protein normalized to GFP. Bar chart shows means ± SDs. Statistical significance was determined using 2-tailed Student’s t test; ^∗^p < 0.05, ^∗∗^p < 0.01, and ^∗∗∗^p < 0.001. (E) Sir2 WB on protein lysates prepared from 7-day-old w1118 and the Sir2[2A- 7-11] mutant. Tubulin was used as a loading control. (F and G) Confocal sections of fat bodies from w1118 (F) and the Sir22A-7-11 (G) larvae stained with LysoTracker Red after 4 h of starvation. Scale bar: 10 μm. (H) CoIP of Sir2-myc and GFP-Atg8a from extracts of fed and starved larvae. Sir2-myc was immunoprecipitated from larva lysates and subjected to SDS-PAGE. (I) Quantification of the relative quantity of GFP-Atg8a co-precipitated with Sir2-myc. Bars denote means ± SDs. Statistical significance was determined using 1-way ANOVA; ^∗∗∗^p < 0.001. (J) *In vitro* interaction between recombinant GST-Atg8a and Sir2-myc expressed in larvae. GST, GST-Atg8a-WT (W, wild-type) or GST-Atg8a-LDS (L, LDS mutated) were pulled down and incubated with larval lysate expressing Sir2-myc. Eluates were subjected to SDS-PAGE. Membrane was stained with Ponceau S for total protein staining and immunoblotted with anti-myc antibody for Sir2-myc. (K) Quantification of the relative quantity of Sir2-myc co-precipitated with GST recombinant proteins. Bars denote means ± SDs. Statistical significance was determined using 1-way ANOVA; ^∗∗∗∗^p < 0.0001. (L) GST-pull-down assay between GST-tagged Atg8a-WT; the acetylation mutants K46Q, K47Q, and K48Q; the LDS mutant (Y49A); and radiolabeled ^35^S-labeled Sequoia-WT or ^35^S-labeled Sequoia-LIR mutant. (M) Quantifications of the binding of radiolabeled myc-Sequoia-WT or LIR mutant (Y313A/I316A) to GST-ATG8a-WT or the respective mutants represented as percentage binding relative to 10% of the input. The bars represent the mean values with SDs from 3 independent experiments. The statistical significance of the Sequoia binding with Atg8a-WT compared to its mutation K48Q was determined with Student’s t test; p = 0.00192. Genotypes for C and D: Control RNAi: Cg-GAL4 UAS-GFP-Atg8a/+;UAS-luc-RNAi/+, YL-1-RNAi: Cg-GAL4 UAS-GFP-Atg8a/+;UAS-YL-1-RNAi/+, Sir2-myc: Cg-GAL4 UAS-GFP-Atg8a/+;UAS-Sir2-myc/+, GFP: Cg-GAL4/+;UAS-GFP/+.

In a bioinformatics screening for Atg8-interacting proteins, we identified YL-1 (CG4621), a subunit of a nuclear acetyltransferase complex, as a putative interactor of Atg8a. YL-1 belongs to the multi-subunit chromatin-remodeling complexes called SWR1 in yeast and the related SRCAP and NuA4/Tip60 complexes in mammals that have been shown to control histone acetylation (Cai et al., 2005, Flegel et al., 2016, Kusch et al., 2004, Latrick et al., 2016, Liang et al., 2016) and have been previously shown to regulate acetylation of autophagy-related proteins Atg3 and ULK1 in yeast and mammals (Lin et al., 2012, Yi et al., 2012). Tip60 has acetyltransferase activity, whereas YL-1 has a regulatory role (Cai et al., 2005, Flegel et al., 2016, Kusch et al., 2004, Latrick et al., 2016, Liang et al., 2016). We therefore sought to explore the role of YL-1 in autophagy in *Drosophila*. *In vitro* translated YL-1 (^35^S-Myc-YL-1) bound very strongly and directly to the N-terminal half (amino acid residues 1–71) of recombinant GST-Atg8a (Figures 4A and 4B). However, mutation of either the LDS of Atg8a (Figures 4A and 4B) or the putative LIR motifs of YL-1 (Figure S2E) did not abrogate significantly the interaction between Atg8a and YL-1, suggesting that this interaction is likely to be LIR motif independent. Since constructs of Atg8a harboring residues 26–121 or only residues 1–26 do not bind to YL-1 (Figure 4B), the N-terminal 26 amino acids of Atg8a are required for binding but are not sufficient. In addition, transgenic flies expressing GFP-YL-1 exhibit nuclear localization, confirming a nuclear role for YL-1 (Figure S2F). We also found that human YL-1/VPS72 interacts with GABARAP (Figure S1B). Since YL-1 is a component of the NuA4/Tip60 acetyltransferase complex, we examined whether YL-1 regulates the acetylation of Atg8a. To examine this, we immunoprecipitated GFP-Atg8a and used an anti-acetyl-lysine antibody to reveal Atg8a acetylation by western blotting. We found that GFP-Atg8a is acetylated in fed conditions and that its acetylation is reduced after starvation and when YL-1 is depleted using RNAi (Figures 4C, 4D, and S2G). These data show that YL-1 is a novel Atg8a-interacting protein and regulates the acetylation of Atg8a.

### Sir2 Interacts with and Deacetylates Atg8a during Starvation

To further investigate the impact of acetylated Atg8a on the activation of autophagy, we focused on the deacetylase Sir2, a homolog of mammalian Sirtuin-1, that has been shown to play a role in lipid metabolism and insulin resistance (Banerjee et al., 2012, Palu and Thummel, 2016, Reis et al., 2010). The Sir2 homolog in mice has been shown to directly deacetylate autophagy machinery components, so we sought to investigate its role in *Drosophila* (Lee et al., 2008). Using a fly line for the expression of myc-tagged Sir2 (Sir2-myc), we found that Sir2 localizes in the nucleus of fat body cells in both fed and starved larvae (Figures S3A and S3B). Fat bodies clonally overexpressing Sir2-myc and stained for acetylated lysine revealed a reduction in nuclear staining for acetylated protein in clonal cells compared to their WT neighbors (Figures S3C–S3E). This suggests that dSir2 is required for deacetylation in the nucleus.

Staining fat bodies from *Sir2* mutant starved larvae for endogenous Atg8a or LysoTracker Red failed to show an accumulation of autophagosomes and autolysosomes in fat body cells (Figures 4E–4G and S3F). Furthermore, Sir2 mutants showed a decrease in Atg8a lipidation during starvation (Figures S4A and S4B). In addition, the overexpression of Sir2-myc resulted in a reduction in GFP-Atg8a acetylation and an increase in Atg8a lipidation in fed and starved larvae (Figures 4C, 4D, S4C, and S4D). GFP-Atg8a cleavage was also increased in starved larvae (Figure 4C). In the same settings, YL-1 depletion using RNAi showed a moderate increase in Atg8a lipidation (Figures S2A and S2B). Moreover, we observed that Sir2-myc interacted preferentially with GFP-Atg8a in starved conditions (Figures 4H and 4I). Using an *in vitro* GST-pull-down assay, we showed that Sir2 interacted preferentially with WT Atg8a compared to an Atg8a-LDS mutant (Figures 4J and 4K). These results suggest that deacetylation of Atg8a by Sir2 is required for the activation of autophagy during starvation.

### Effect of Acetylation of Atg8a on Binding to Sequoia

We next investigated whether the interaction of Atg8a with Sequoia is regulated by the acetylation status of Atg8a in fed conditions (when Atg8a is acetylated) and in starved conditions (when Atg8a is deacetylated). To test this, we examined the binding of WT and acetylation mimic forms of Atg8a to Sequoia. LC3 has been shown to be acetylated at residues K49 and K51 in fed conditions (Huang et al., 2015). Therefore, we tested the homologous residues K46 and K48 in *Drosophila* Atg8a. We found that only acetylation mimic mutation Atg8a K48Q showed a significant decrease in its binding to Sequoia (Figures 4L and 4M). To examine the effect of K48 acetylation on binding to Sequoia, we also created a homology model of the LIR peptide of Sequoia binding to *Drosophila* Atg8a based on the structure of GABARAP-L1 ATG4B LIR complex (PDB: 5LXI) (Figures S4E and S4F). The residues Y313 and I316 of the LIR peptide of Sequoia are likely to bind in the HP1 and HP2 pockets, respectively. The negatively charged glutamates (E) will interact with the positively charged lysine (K) residues of the 44-LDKKKYLVP-52 motif (shown as sticks under the surface). Upon acetylation, the K48 residue will become bulkier, will lose the positive charge, and the potential salt-bridge interaction between K48 and E309 will be removed by acetylation. These data suggest that deacetylated Atg8a during starvation binds more strongly to Sequoia.

### Atg8a Regulates the Expression of Autophagy Genes during Starvation

All of the above results show a role for Atg8a-interacting proteins Sequoia, YL-1, and Sir2 in the expression of autophagy genes. To examine the direct role of Atg8a on autophagy gene expression, we performed real-time qPCR experiments in Atg8a mutants in which the Atg8a protein is not present. We observed that the absence of Atg8a has a significant negative impact on the expression of other autophagy genes during starvation (Figure S5A). However, lack of Atg9, which is part of a different complex of autophagy proteins required for the initiation of autophagosome formation, demonstrated very little or no impact on the expression of autophagy genes (Figure S5B). Furthermore, using a ChIP assay, we observed that Atg8a is associated with the promoter region of autophagy genes (Figure S5C). These results suggest a role for Atg8a in the regulation of the expression of autophagy genes during starvation.

## Discussion

Atg8 family proteins have been extensively described for their implications in autophagosome formation and cargo selection in the cytoplasm. Although Atg8 family proteins also localize in the nucleus, their role in this compartment remains largely unexplored. Here, we uncovered a nuclear role for *Drosophila* Atg8a in the regulation of autophagy gene expression and induction of autophagy via a LIR motif-dependent mechanism, regulated by Atg8a acetylation. We demonstrated that the transcription factor Sequoia interacts with Atg8a in the nucleus to control the transcriptional activation of autophagy genes. We suggest that the acetylation status of Atg8a at position K48 contributes to the modulation of the interaction between Sequoia and Atg8a in the nucleus. We also identified that YL-1, a component of a nuclear acetyltransferase complex, and deacetylase Sir2 interact with Atg8a, and that they act as regulators of Atg8a acetylation.

We propose a working model in which in fed conditions, histone-interacting protein YL-1 contributes to the acetylation of Atg8a, while Sequoia resides at the promoter regions of autophagy genes to repress their expression (Figure S5D). In such conditions, the interaction between Sequoia and Atg8a contributes to the sequestration of Atg8a in the nucleus. Atg8a cannot therefore translocate to the cytoplasm to take part in the formation of autophagosomes. This hypothesis is supported by the observation that mutation of the Sequoia LIR motif results in an increased accumulation of autophagosomes and autolysosomes in the fed condition (Figure S5D). This is observed alongside a reduction in the enrichment of Sequoia at the promoter region of autophagy genes, resulting in their increased expression. Hence, in the absence of an interaction between Atg8a and Sequoia, the subsequent translocation of Atg8a to the cytoplasm may also play a key role in relieving the repressive abilities of Sequoia at the promoter regions of autophagy genes. Upon starvation, Sir2 interacts with and deacetylates Atg8a. Deacetylated Atg8a interacts more strongly with Sequoia, which cannot be maintained at the promoter regions of autophagy genes, leading to the activation of their transcription. Deacetylated Atg8a is then able to translocate to the cytoplasm and contribute to the formation of autophagosomes (Figure S5D). We propose that Atg8a plays an essential role in relieving Sequoia from the promoter regions of autophagy genes specifically during starvation-induced autophagy as Atg8a loss of function results in the repression of the expression of autophagy genes (Figure S5D).

Our results support previous findings about the yeast and mammalian homologs of Sequoia, Rph1, and KDM4A, respectively, which have been shown to negatively regulate the transcription of autophagy genes (Bernard et al., 2015). Here, we elucidate how the LIR-dependent interaction between Sequoia and Atg8a is involved in modulating the expression of autophagy genes during starvation. Mammalian KDM4A also directly interacts with GABARAP-L1; however, the interaction does not require a functional LIR motif. This may be related to the loss of the functionality of the LIR motif during evolution, as it has been shown for Kenny, another LIR-motif containing protein in *Drosophila*, and its mammalian homolog inhibitor of nuclear factor κB kinase (NF-κB) subunit γ/NF-κB essential modulator (IKKγ/NEMO) (Tusco et al., 2017). Our study also supports previous reports about the role of acetylation and deacetylation of LC3 in mammals (Huang et al., 2015, Lee et al., 2008, Lee and Finkel, 2009, Li et al., 2016, Song et al., 2019) and the regulation of autophagy by acetylation (Flegel et al., 2016, Li et al., 2017).

Higher eukaryotes express YL-1, a highly conserved Swc2 homolog, which has specific H2A.Z-binding properties. *Drosophila* YL-1 has been shown to have a H2A.Z-binding domain that binds H2A.Z-H2B dimer (Liang et al., 2016). Here, we report a novel role for YL-1 in the regulation of acetylation of non-histone proteins and the regulation of autophagy induction.

In conclusion, our results unveil a novel nuclear role for Atg8a in the regulation of autophagy gene expression in *Drosophila*, which is linked to its acetylation status and its ability to interact with transcription factor Sequoia. Our study highlights the physiological importance of the non-degradative role of LIR motif-dependent interactions of Atg8a with a transcription factor and provide novel mechanistic insights on an unanticipated nuclear role of a protein that controls cytoplasmic cellular self-eating.

## STAR★Methods

### Key Resources Table

**Table:** 

REAGENT or RESOURCE	SOURCE	IDENTIFIER
**Antibodies**		

anti-GFP	Abcam	ab290; RRID:AB_303395
anti-myc	Cell Signaling Technology	#2276; RRID:AB_331783
anti-mCherry	Novus NBP1	#96752; RRID:AB_11034849
anti-alpha tubulin	Sigma-Aldrich	T5168; RRID:AB_477579
anti-acetyl lysine	Cell Signaling	#9441; RRID:AB_331805
anti-dSir2	DSHB	p4A10; RRID:AB_1553778
anti-mouse HRP	Thermo Scientific	#31450, #31460; RRID:AB_228341
Veriblot HRP-coupled for IP detection	Abcam	ab131366
anti-Cathepsin-L	Abcam	ab58991; RRID:AB_940826
Normal Rabbit IgG	Cell Signaling	#2729; RRID:AB_1031062
anti-Sequoia	Gift from Prof. Y Jan	N/A
Histone H3 Antibody	Cell Signaling	#9715; RRID:AB_331563
anti-GABARAP	Abcam	ab109364; RRID:AB_10861928
anti-Atg1	Gift from Gabor Juhasz, Eotvos Lorand University, Budapest. (Nagy et al., 2014)	N/A
a-actin	Abcam	ab8227; RRID:AB_2305186

**Bacterial and Virus Strains**		

BL21(DE3) Competent *E.Coli*	New England Biolabs	Cat#C2527I
SoluBL21™ Competent *E.Coli*	Ambio	C700200
Rosetta2(DEM3) Singles Competent Cells	Novagen	71400

**Chemicals, Peptides, and Recombinant Proteins**		

EDTA-free protease inhibitor cocktails	Roche	5892791001
Deactylation inhibitors	Santa Cruz	sc-362323
Sepharose-coupled G-beads	Sigma-Aldrich	Cat#28-9670-66
LysoTracker™ Deep Red	Thermo Fisher Scientific	L12492
Glutathione-Sepharose 4 Fast Flow beads	Amersham Biosciences	Cat#17513201
Formaldehyde	Sigma-Aldrich	Cat#F8775
Protein A beads	GE Healthcare	28967062
GoTaq qPCR Master Mix	Promega	A6002

**Critical Commercial Assays**		

PureLink™ RNA mini kit	Life Technologies	Cat#12183025
RevertAid Kit	Thermo Scientific	Cat#K1622
QuickChange site-directed mutagenesis	Stratagene	200523
Phusion High-Fidelity DNA Polymerase	Thermo Scientific	Cat#F-530XL
EasyTag™ L-[35S]-methionine	PerkinElmer Life Sciences	NEG709A500UC

**Experimental Models: Organisms/Strains**		

*w*^*1118*^	Bloomington *Drosophila* stock center	#3605
*Cg-GAL4*	Bloomington *Drosophila* stock center	#7011
*UAS-YL-1-RNAi*	Bloomington *Drosophila* stock center	#31938
*UAS-Sir2-myc*	Bloomington *Drosophila* stock center	#44216
*UAS-Sequoia-RNAi*	Vienna Drosophila Resource Centre	#50146
*Atg7*^*Δ77*^	Gift from Gabor Juhasz, Eotvos Lorand University, Budapest. (Juhász et al., 2007)	N/A
*Atg7*^*Δ14*^	Gift from Gabor Juhasz, Eotvos Lorand University, Budapest. Juhász et al., 2007)	N/A
*Sir2*^*2A-7-11*^	Bloomington *Drosophila* stock center	#8838
*yw hs-flp; UAS-mCherry-Atg8a;Ac > CD2 > GAL4*	Tusco et al. (2017)	N/A
*UAS-GFP-Sequoia-WT*	This paper; generated by P-element-mediated transformation (BestGene Inc)	N/A
*UAS-GFP-Sequoia-LIRm (Y313A/316A)*	This paper; generated by P-element-mediated transformation (BestGene Inc)	N/A
*UAS-GFP-YL-1*	This paper; generated by P-element-mediated transformation (BestGene Inc)	N/A
*Atg9*^*B5*^*/CyO*	Gift from Gabor Juhasz, Eotvos Lorand University, Budapest. (Kiss et al., 2020)	N/A
*Atg9*^*DF(ED2487)*^*/GFP twi CyO*	Gift from Gabor Juhasz, Eotvos Lorand University, Budapest. (Kiss et al., 2020)	N/A
Atg8a [KG07569]	(Scott et al., 2007)	N/A
*UAS-Sequoia-RNAi*	Bloomington *Drosophila* stock center	#51923

**Oligonucleotides**		

Primers for mRNA Atg expression, see Table S1	This paper	N/A
Primers for Atg promotor region, see Table S1	This paper	N/A

**Recombinant DNA**		

Plasmid: pPGW	*Drosophila* Genomics Resource Centre	1077
Gateway pDONR221 Vector	Thermo Scientific	Cat#12536017
Gateway™ pENTR™	Thermo Scientific	SKU#A10467

**Software and Algorithms**		

iLIR database	Kalvari et al. (2014)	http://repeat.biol.ucy.ac.cy/iLIR
AtgCOUNTER (ImageJ/Fiji macro)	Jacomin and Nezis (2016)	https://imagej.nih.gov/ij/
PyMol	The PyMOL Molecular Graphics System, Version 2.0 Schrödinger, LLC.	https://pymol.org/2/

### Resource Availability

#### Lead Contact

Additional information and requests for reagents and protocols should be directed to and will be fulfilled by the Lead Contact, Ioannis Nezis (I.Nezis@warwick.ac.uk).

#### Materials Availability

All materials are publicly available. Please contact Dr Ioannis Nezis.

#### Data and Code Availability

The published report includes all data generated or analyzed during this study. No code was used or generated during this study.

### Experimental Model and Subject Details

#### Fly Husbandry and Generation of Transgenic Lines

Flies used in experiments were kept at 25°C and 70% humidity raised on cornmeal-based feed. The following fly stocks were obtained from the Bloomington *Drosophila* stock center: *w*^*1118*^ (#3605), *Cg-GAL4* (#7011) and *UAS-YL-1-RNAi* (#31938), *UAS-Sir2-myc (#44216)*, *UAS-Sequoia-RNAi* (#51923). *UAS-Sequoia-RNAi* (#50146) was obtained from the Vienna *Drosophila* Resource Center. The following mutant lines have been used: *Atg7*^*Δ77*^, and *Atg7*^*Δ14*^*/CyO-GFP* (Juhász et al., 2007), *Atg9*^*B5*^*/CyO* and *Atg9*^*DF(ED2487)*^*/GFP twi CyO* (Kiss et al., 2020), Atg8a [KG07569] (Scott et al., 2007), *Sir2*^*2A-7-11*^ from Bloomington (#8838). The clonal analysis using the FLPout system has been performed with the following lines: *yw hs-flp; UAS-mCherry-Atg8a;Ac > CD2 > GAL4*. The transgenic lines UAS-GFP-Sequoia-WT, UAS-GFP-Sequoia-LIRm (Y313A/316A) and UAS-GFP-YL-1 have been generated by cloning the cDNA of *sequoia* or *YL-1* respectively into the pPGW plasmid (DGRC). Transgenic flies were generated by P-element-mediated transformation (BestGene Inc). Early third-instar larvae were collected either fed or starved for 4 hours in 20% sucrose solution in PBS.

### Method Details

#### Protein Extraction, Immunoprecipitation, and Western Blotting

Protein content was extracted from larvae in Nuclear lysis buffer (20 mM Tris pH 7.5, 137 mM NaCl, 1mM MgCl_2_, 1mM CaCl_2_, 1% Igepal, 10% Glycerol, 1mM Na_3_VO_4_, 15mM Na_4_P_2_O_7_, 5mM Sodium butyrate supplemented with EDTA-free proteases inhibitors cocktail (Roche, 5892791001) and deacetylase inhibitors (Santa Cruz, sc-362323) using a motorized mortar and pestle. Co-immunoprecipitations were performed on lysates from flies expressing GFP alone, GFP-Atg8a or Sir2-myc along with mCherry-Atg8a. After a 30 min pre-clear of the lysates (1 mg total proteins) with Sepharose-coupled G-beads (Sigma), the co-immunoprecipitations were performed for 2 h at 4°C using an anti-GFP antibody (Abcam, Ab290) or anti-myc (Cell Signaling Technology, #2276) and fresh Sepharose-coupled G-beads. Four consecutive washes with the lysis buffer were performed before suspension of the beads in 60 μL 2X Laemmli loading buffer. All protein samples (whole fly lysates and co-immunoprecipitation eluates) were boiled for 5-10 min at 95°C. Quantity of 10–40 μg of proteins (whole lysates) or 20 μL (co-immunoprecipitation eluates) were loaded on polyacrylamide gels and were transferred onto either nitrocellulose or PVDF membranes (cold wet transfer in 10%–20% ethanol for 1h at 100V). Membranes were blocked in 5% BSA or non-fat milk in TBST (0.1% Tween-20 in TBS) for 1 h. Primary antibodies diluted in TBST were incubated overnight at 4°C or for 2 h at room temperature with gentle agitation. HRP-coupled secondary antibodies binding was done at room temperature (RT) for 45 min in 1% BSA or non-fat milk dissolved in TBST and ECL mix incubation for 2 min. All washes were performed for 10 min in TBST at RT. The following primary antibodies were used: anti-GFP (Santa Cruz sc-9996, 1:1000), anti-mCherry (Novus NBP1 #96752, 1:1000), anti-alpha tubulin (Sigma-Aldrich T5168, 1:50,000), anti-acetyl lysine (Cell Signaling #9441, 1:1000), anti-dSir2 (DSHB p4A10, 1:50), anti-Atg1 (Nagy et al., 2014). HRP-coupled secondary antibodies were from Thermo Scientific (anti-mouse HRP #31450; anti-rabbit HRP #31460). Following co-immunoprecipitation, Veriblot HRP-coupled IP secondary antibody was used (Abcam ab131366, 1:5000).

#### Immunohistochemistry

Larva tissues were dissected in PBS and fixed for 30 min in 4% formaldehyde in PBS. Blocking and antibody incubations were performed in PBT (0.3% BSA, 0.3% Triton X-100 in PBS). Primary and secondary antibodies were incubated overnight at 4°C in PBT. The following primary antibody was used: anti-Cathepsin-L (Abcam ab58991, 1:400). Washes were performed in PBW (0.1% Tween-20 in PBS). All images were acquired using Carl Zeiss LSM710 or LSM880 confocal microscopes, using a × 63 Apochromat objective. Staining with LysoTracker was performed by incubating the non-fixed larval fat body in LysoTracker Red in PBS (1:1,000) for 10 min followed by mounting in 75% glycerol in PBS.

#### Real-Time qPCR

RNA extraction was performed with a Life Technologies Ambion PureLinkTM RNA Mini kit according to the manufacturer protocol. Fat bodies from 20 L3 larvae were used per extract. Subsequent steps were performed using 1 μg of total RNA. ThermoScientific DNase I was used in order to digest genomic DNA. The ThermoScientific RevertAid Kit was subsequently used to synthesize cDNA. RT-qPCR was performed using the Promega GoTaq qPCR Master Mix (ref. A6002). Primer sequences are available in Table S2.

#### Plasmid Constructs

Sequences of the genes of interest were amplified by PCR and inserted in desired plasmid using either Gateway recombination system or restriction enzyme cloning. PCR products were amplified from cDNA using Phusion high fidelity DNA polymerase with primers containing the Gateway recombination site or restriction enzyme sites for Gateway entry vector and cloned into pDONR221 or pENTR using Gateway recombination cloning. Point mutants were generated using the QuikChange site-directed mutagenesis (Stratagene, 200523). Plasmid constructs were verified by conventional restriction enzyme digestion and/or by DNA sequencing.

#### GST Pull-Down Assays

GST-fusion proteins were expressed in *Escherichia coli* BL21(DE3), SoluBL21 or Rosetta2. GST-fusion proteins were purified on glutathione-Sepharose 4 Fast Flow beads (Amersham Biosciences). GST pull-down assays were performed using either recombinant proteins produced in bacteria or *in vitro* translated ^35^S-methionine-labeled proteins. L-[35S]-methionine was obtained from PerkinElmer Life Sciences. A volume of 10 μL of the *in vitro* translation reaction products (0.5 μg of plasmid in a 25 μL reaction volume) were incubated with 1–10 μg of GST-recombinant protein in 200 μL of NETN buffer (50 mM Tris, pH 8.0, 150 mM NaCl, 1 mM EDTA, 0.5% Nonidet P-40, 1 mM dithiothreitol supplemented with Complete Mini EDTA-free protease inhibitor cocktail (Roche Applied Science)) for 2 h at 4°C, washed six times with 1 mL of NETN buffer, boiled with 2X SDS gel loading buffer, and subjected to SDS-PAGE. Gels were stained with Coomassie Blue and vacuum- dried. ^35^S-Labeled proteins were detected on a Fujifilm bio-imaging analyzer BAS- 5000 (Fuji).

#### Chromatin Immunoprecipitation Assay

Approximately 200 whole larvae from wild-type (WT) flies were fixed with 1% Formaldehyde (Sigma-Aldrich, Cat#: F8775) at 37°C for 15 min followed by incubation on ice for 2 min. For the supernatant preparation 200 μl of lysis buffer was added (50 mM Tris-HCl, pH 7.6, 1 mM CaCl_2_, 0.2% Triton X-100 or NP-40, 5 mM butyrate, and 1X proteinase inhibitor cocktail). The lysates were subjected to sonication to shear DNA to the length of approximately between 150 and 900 bp using an Epishear™ Probe Sonicator (Active motif). The sample was then diluted by adding RIPA buffer (10 mM Tris-HCl, pH 7.6, 1 mM EDTA, 0.1% SDS, 0.1% Na-Deoxycholate, 1% Triton X-100, supplemented with protease inhibitors and PMSF). For the input samples 40 μl were saved and supplemented with 2 μl 5M NaCl and were incubated at 65°C O/N to reverse crosslink. The rest of the lysate was then incubated with control IgG (Cell Signaling Technology, Cat#: 2729S) or primary antibody against Sequoia (gift from Prof Y Jan) together with 40 μl of Protein A beads (GE Healthcare, 28967062) at 4°C O/N. The beads were washed sequentially with the following buffers: RIPA buffer, RIPA buffer supplemented with 0.3M NaCl, LiCl buffer (0.25 M LiCl, 0.5% NP40, 0.5% NaDOC), TE buffer supplemented with 0.2% Triton X-100 and TE buffer. To reverse crosslink, the beads were resuspended in 100 μl TE buffer (supplemented with 10% SDS and Proteinase K (20 mg/ml) and were incubated at 65°C O/N. The DNA was purified by phenol-chloroform extraction followed by qPCR analysis. Primers are listed in Table S2, promotor region sequences are listed in Table S1.

#### Structural Modeling

The model of the peptide of Sequoia binding to *Drosophila* ATG8a was derived from the structure of GABARAP-L1 ATG4B LIR Complex (PDB code 5LXI). Essentially all residues in the structure were mutated to the correct sequence. No energy minimization was carried out. The structures and electrostatic surfaces were displayed in PyMol. As an approximation the electrostatic surface associated with the acetylated protein was calculated with a methionine as a mimic of the acetylated K48.

### Quantification and Statistical Analysis

#### Quantification of mCherry-Atg8a Puncta

Fiji/ImageJ was used to quantify the mCherry-Atg8a dots using the macro AtgCOUNTER (Jacomin and Nezis, 2016).

#### Quantification of Binding Interaction Assays

Signals from ^35^S-labeled proteins were measured in terms of unit of photostimulated luminescent (PSL) and quantitated in comparison with 10% of the *in vitro*-translated lysate using the Image Gauge software (Fuji).

#### Statistical Analysis

Statistical analysis was performed as described in figure legends. All statistical analysis was performed with GraphPad Prism7, along with graph generation.

## Supplemental Information

Document S1. Figures S1–S5 and Table S1

Document S2. Article plus Supplemental Information
