# Supplementary material for: Regulation of Expression of Autophagy Genes by Atg8a-Interacting Partners Sequoia, YL-1, and Sir2 in Drosophila
Source: Cell Rep. 2020 May 26;31(8):107695. doi: 10.1016/j.celrep.2020.107695 (PMC7262597; doi:10.1016/j.celrep.2020.107695)
Supplement: Document S1. Figures S1–S5 and Table S1 [file mmc1.pdf]

Cell Reports, Volume *31*

Supplemental Information

Regulation of Expression of Autophagy Genes by Atg8a-Interacting Partners Sequoia, YL-1, and Sir2 in *Drosophila*

Anne-Claire Jacomin, Stavroula Petridi, Marisa Di Monaco, Zambarlal Bhujabal, Ashish Jain, Nitha C. Mulakkal, Anthimi Palara, Emma L. Powell, Bonita Chung, Cleidiane Zampronio, Alexandra Jones, Alexander Cameron, Terje Johansen, and Ioannis P. Nezis

Supplemental Information

Regulation of expression of autophagy genes by Atg8a-interacting partners Sequoia, YL-1 and Sir2 in *Drosophila*

Anne-Claire Jacomin, Stavroula Petridi, Marisa Di Monaco, Zambarlal Bhujabal, Ashish Jain, Nitha C. Mulakkal, Anthimi Palara, Emma L. Powell, Bonita Chung, Cleidiane Zampronio, Alexandra Jones, Alexander Cameron, Terje Johansen and Ioannis P. Nezis

A Submitted 1 sequence
>tr|A1Z995|A1Z995_DROME Sequoia, isoform A OS=Drosophila melanogaster GN=seq PE=4 SV=1

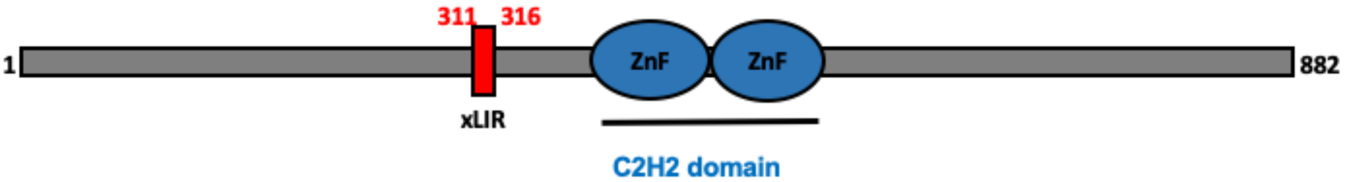

xLIR motif is displayed as red rectangles in the domain architecture graphic

MOTIF	START	END	LIR Sequence	PSSM Score	Anchor
xLIR	311	316	EEYQVI	14 (5.7e-02)	Yes
WxxL	167	172	QHYHYI	3 (1.9e+00)	Yes
WxxL	237	242	EEYHVL	11 (1.5e-01)	Yes
WxxL	392	397	ARYEHI	9 (2.8e-01)	No
WxxL	401	406	VAYCLV	1 (3.6e+00)	No
WxxL	728	733	QSYQJL	11 (1.5e-01)	Yes
WxxL	818	823	QQFMEL	6 (7.4e-01)	Yes

B

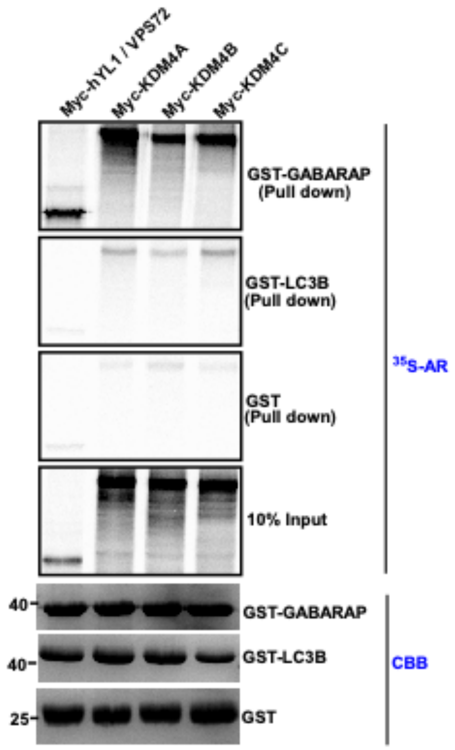

C

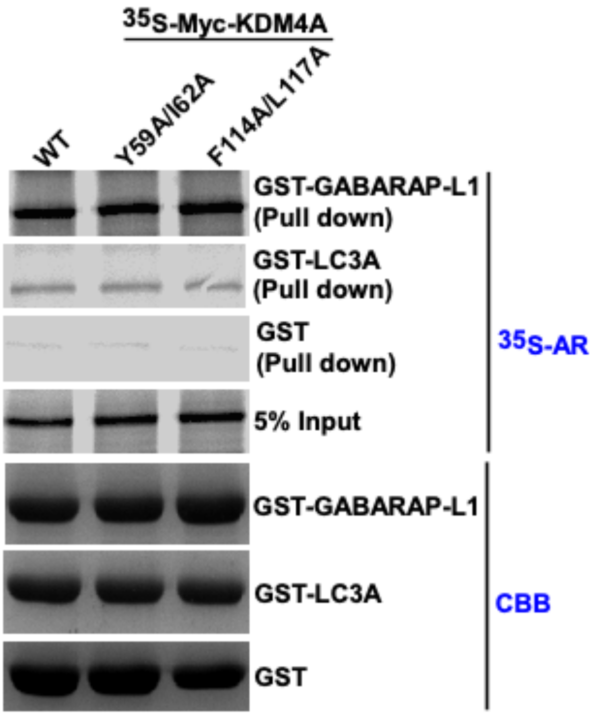

D

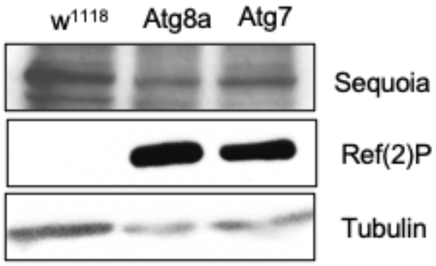

Figure S1 (related to Figure 1)

Figure S1 (related to Figure 1)

(A) Sequoia is a Zinc finger domain-containing protein with a xLIR motif. Screenshot of the result window from the iLIR server (http://ilir.warwick.ac.uk/) for the protein sequence of Sequoia (Uniprot A1Z995). xLIR relaxed LIR motif; WxxL: conventional LIR motif; ZnF: Zinc finger motif; PSSM: position-specific scoring matrix. (B) GST-pull-down assay between GST-tagged GABARAP or LC3B and radiolabelled myc-KDM4A, myc-KDM4B, myc-KDM4C. GST was used as negative control. (C) GST-pull-down assay between GST-tagged GABARAP-L1 or LC3A and radiolabelled myc-KDM4A-WT or -LIR mutant (Y59A/I62A) or –LIR mutant (F114A/L117A). GST was used as negative control. (D) Whole body lysates from wild-type (WT) and Atg8a and Atg7 mutant flies were subjected to SDS-PAGE and immunoblotting for Sequoia and Ref(2)P. Tubulin was used as loading control. Genotypes: w^{118}, Atg8a, $Atg7^{D14}/Atg7^{D77}$.

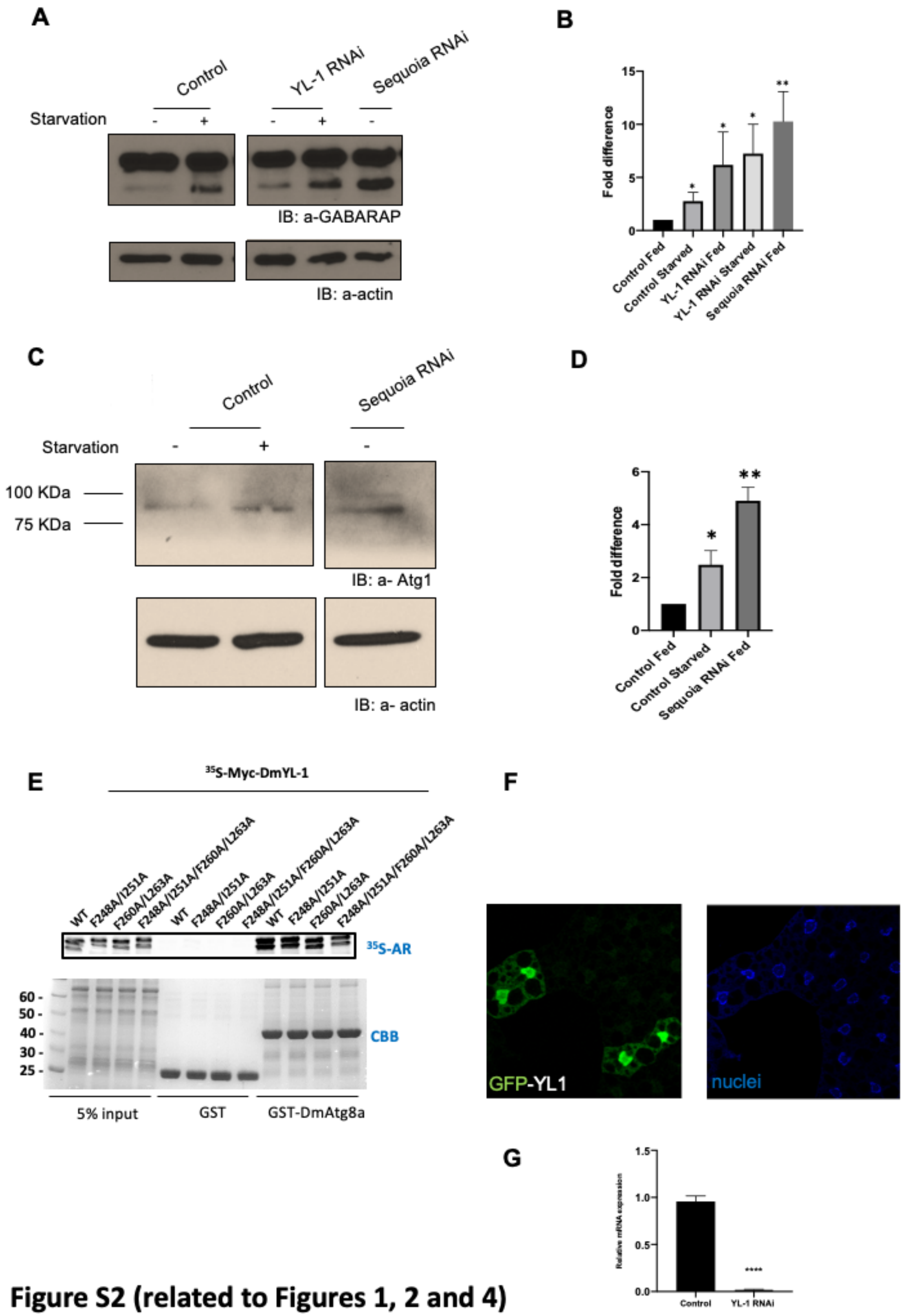

Figure S2 (related to Figures 1, 2 and 4)

Figure S2 (related to Figures 1, 2 and 4)

(A) Larvae that were constitutively expressing the control Luciferase RNAi, the YL-1 RNAi and the Sequoia RNAi in their fat bodies were collected, both in fed and starved conditions (4h in 20% sucrose). The conversion of Atg8a-I to Atg8a-II was determined by western blotting for an anti-GABARAP antibody. (B) Quantification of the quantity of Atg8a-II protein normalized to actin. Bar chart shows means ± s.d. Statistical significance was determined using two-tailed Student's t-test, *P < 0.05, **P < 0.01, ***P < 0.001. (C) Larvae that were expressing the control Luciferase RNAi and the Sequoia RNAi in their fat bodies were collected, both in fed and starved conditions (4h in 20% sucrose). Atg1 antibody was used to test the expression of endogenous Atg1 by western blotting. (D) Quantification of the quantity of Atg1 protein normalized to actin. Bar chart shows means ± s.d. Statistical significance was determined using two-tailed Student's t-test, *P < 0.05, **P < 0.01. (E) GST-pull down assay between GST-tagged Atg8a-WT, and radiolabelled 35S-YL-1-myc both in WT and different YL-1 LIR motif mutants. (F) Confocal section of larvae fat body cells expressing GFP-YL-1 (green) in fed conditions. (G) Analysis of mRNA expression levels of YL-1 in YL-1 RNAi in comparison to control. YL-1 specific primer pairs were used, with results being showed across 3 technical repeats. Student's t test was performed to check for the statistical significance of differences between YL-1 mRNA levels (****, P < 0.0001).

Genotypes: (A-D) Control RNAi: Cg-GAL4/+; UAS-luc-RNAi/+, YL-1-RNAi: Cg-GAL4 /+; UAS-YL-1-RNAi/+, Sequoia RNAi: Cg-GAL4/+; UAS-Sequoia-RNAi/+. (F) y w hs-Flp ; Ac>CD2>GAL4 / UAS-GFP-YL-1-WT, (G) ctl: Cg-GAL4 /+ ; UAS-luc-RNAi /+, YL-1-RNAi: Cg-GAL4 /+ ; UAS-YL-1-RNAi.

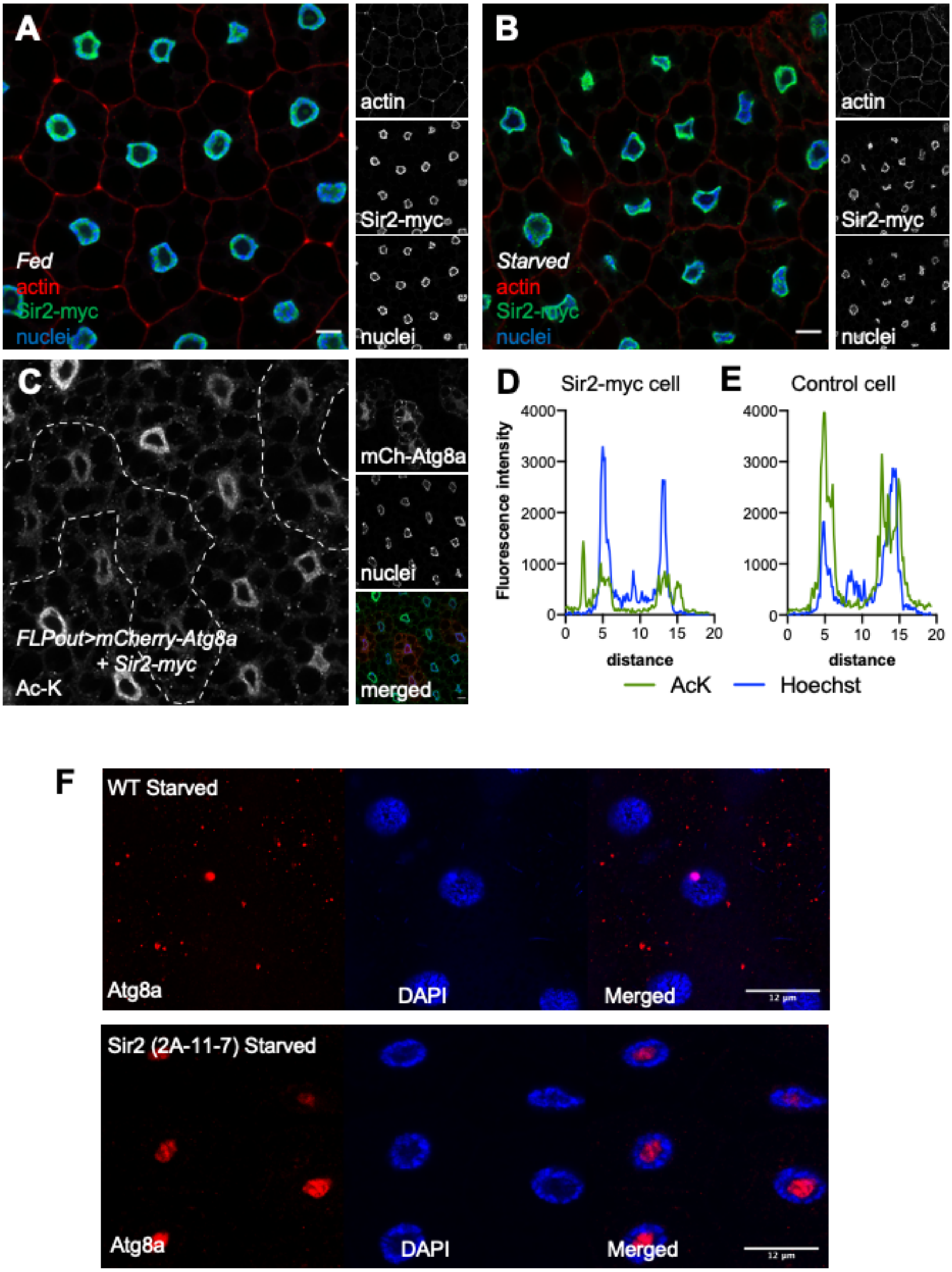

Figure S3 (related to Figure 4)

Figure S3 (related to Figure 4)

(A-B) Confocal sections of fat bodies expressing Sir2-myc (green) from fed (A) and 4hrs starved (B) larvae. Fixed fat bodies where stained nuclei (blue) and cortical actin (red). (C) Confocal sections of larval fat bodies clonally expressing Sir2-myc in combination with the autophagy marker mCherry-Atg8a (red) and stained for acetylated-lysine (green). (D-E) Intensity plots for the green (acetylated-lysine, AcK) and blue (Hoechst) channels taken across nuclei from control (D) or Sir2-myc clonal (E) cells. (F) Confocal sections of larval WT and null Sir2 mutant fat bodies in starved conditions after staining with a-GABARAP (red). Scale bar: 12μm.

Genotypes: (A-B) Cg-GAL4/UAS-Sir2-myc, (C-D) y w hs-Flp ; Ac>CD2>GAL4 / UAS-Sir2-myc ; UAS-mCherry-Atg8a /+, (F) w^{118}, Sir2(2A-11-7).

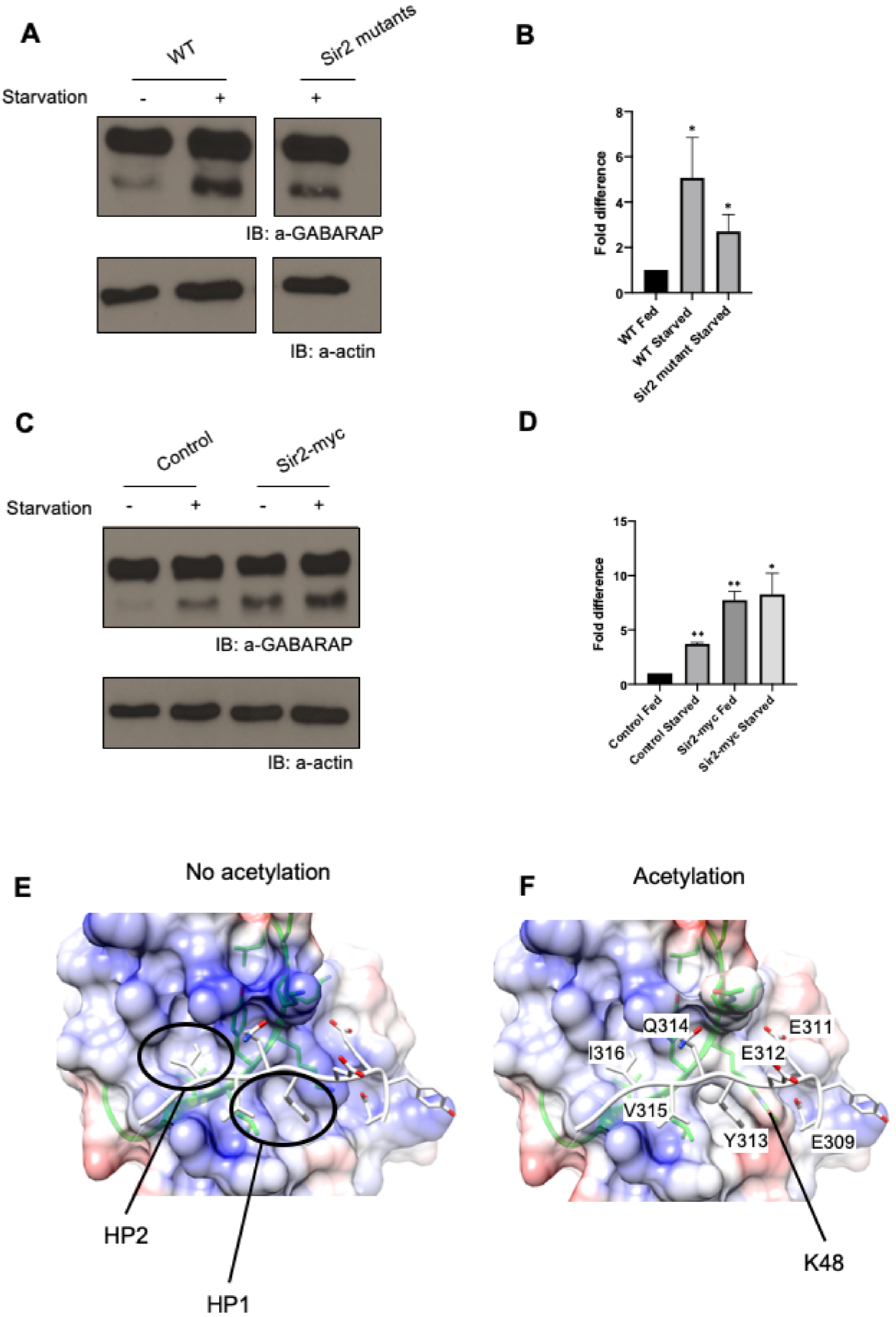

Figure S4 (related to Figure 4)

Figure S4 (related to Figure 4)

Larvae lysates from wild-type (WT) and Sir2 mutant flies were collected, both in fed and starved conditions (4h in 20% sucrose). The conversion of Atg8a-I to Atg8a-II was determined by Western blotting (WB) for an a-GABARAP antibody. (B) Quantification of the quantity of Atg8a-II protein normalized to actin. Bar chart shows means ± s.d. Statistical significance was determined using two-tailed Student's t-test, *P < 0.05, **P < 0.01, ***P < 0.001. (C) Larvae lysates from wild-type (WT) and Sir2-myc flies were collected both in fed and starved conditions (4h in 20% sucrose). The conversion of Atg8a-I to Atg8a-II was determined by Western blotting (WB) for an a-GABARAP antibody. (D) Quantification of the quantity of Atg8a-II protein normalized to actin. Bar chart shows means ± s.d. Statistical significance was determined using two-tailed Student's t-test, *P < 0.05, **P < 0.01, ***P < 0.001. (E-F) Homology model of a peptide of Sequoia binding to *Drosophila* Atg8a LIR complex in starved (E) and fed (F) conditions. Atg8a is represented by a semi-transparent surface coloured according to electrostatic charge; where blue is positive, white is neutral and red is negative. The grey peptide back-bone structure is representative of the Sequoia LIR peptide.

Genotypes: (A) w^{118}, Sir2(2A-11-7), (B) ctl: Cg-GAL4 /+ ; UAS-luc-RNAi /+, Sir2-myc: Cg-GAL4 /Sir2-myc.

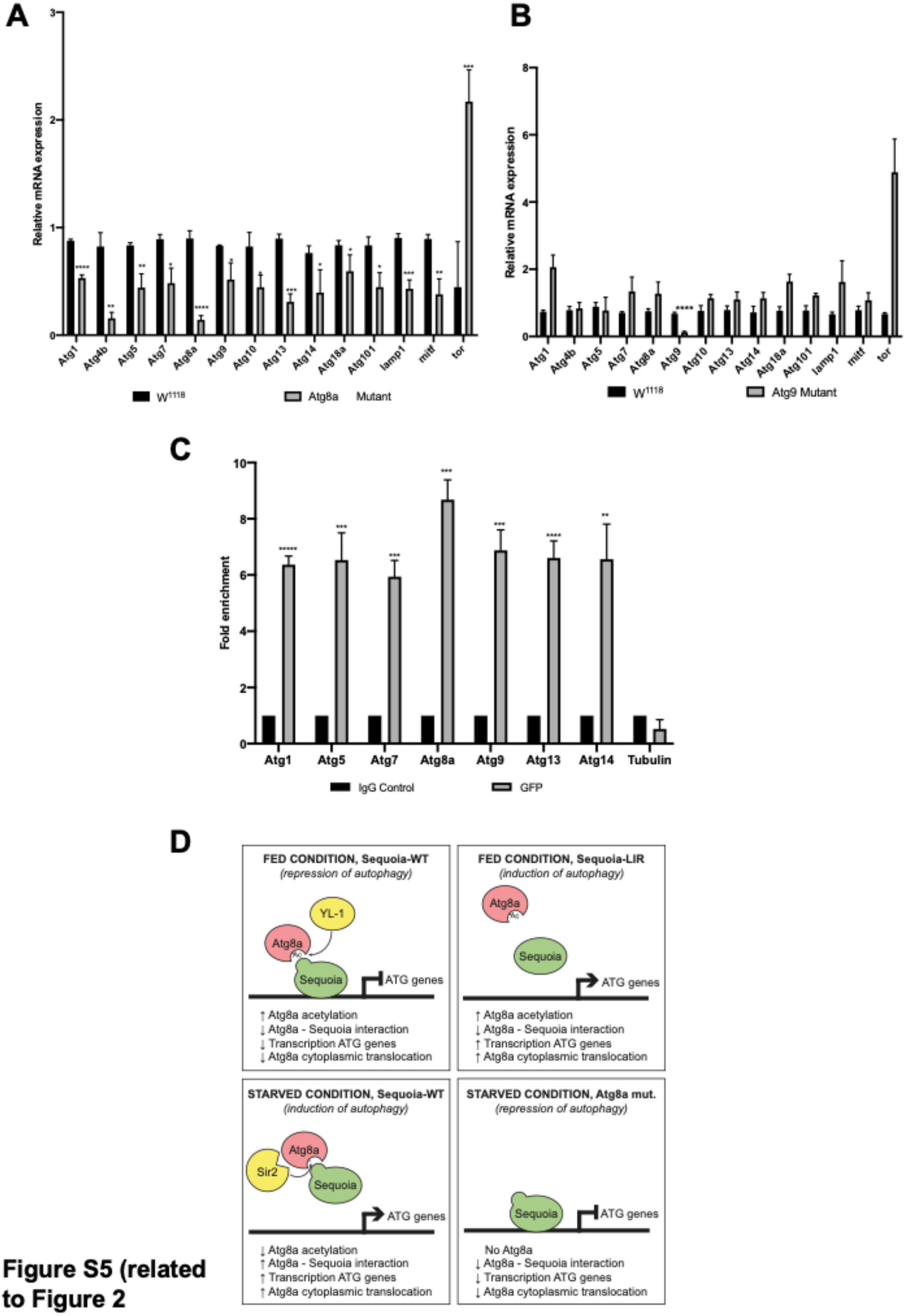

Figure S5 (related to Figure 2

Figure S5 (related to Figure 2)

(A) Analysis of the mRNA levels of autophagy-associated genes in wild-type (w^{1118}) and $Atg8a^{kg}$ null mutant larval fat bodies under starvation conditions, using real time qPCR. (B) Analysis of the mRNA levels of autophagy-associated genes in wild-type (w^{1118}) and homozygous $Atg9^{B5}$ null mutant larval fat bodies under starvation conditions, using real time qPCR. (C) Analyses of Atg8a binding to the promoter of autophagy genes as detected by ChIP (chromatin immunoprecipitation) in fed conditions, using a GFP antibody. ChIP DNA values were normalised to input DNA using the 2-ΔΔCt method. Fold enrichment values are shown relative to the IgG control. Tubulin was used as a non-autophagy related gene control.

All data shown as mean ± SD, n= 3 independent experiments. Statistical significance was determined using Student's *t*-test, *p <0.05- ***p < 0.005. (D) Model of how fed versus starved conditions affect the regulation of autophagy genes by interaction between the transcriptional repressor Sequoia and Atg8a, between Atg8a and the acetyltransferase subunit YL-1, and between Atg8a and the deacetylase Sir2.

Genotypes: (A) Atg8a [KG07569], w^{1118} (B) $Atg9^{B5}/Atg9^{Df(ED2487)}$, w^{1118} (C) Cg-GAL4;UAS-GFP-Atg8a.

Gene symbol	Promotor Region
Atg Genes	
Atg1	ttggcggttcgccacctgcggccacactggccaacccaccacttcgcttAGTTGTGTTTT
Atg5	tgcgatatttcagcgttgccatcttcgaatgccaagtgccatcactattATCAAATAGCA
Atg7	ataagttatcttctcattgtctatcgccatcgAGAGCTTTGTTT
Atg8a	agcgcgtttcgggttggtgtctctgcgcgctcactggtcacacacggtcAGTCTAGCCAC
Atg9	ccttacttattcattacccagtattttgacgttggccaacacttctcttAGGAGAGTCAG
Atg13	cacgcccattatcggactatcgatactatcgcgagtgcTAGCTGACGCT
Atg14	gacatctatttttcgatatcagcctatcgtttagcaataggtgtcctcATATCGATAGC
Non-Atg Control Gene	
β-Tubulin	tggccacactgcggccatcgtataaaagcccgcgctctccaaagcgaatGCACTAATTTT
Rpl30	gtcacaccaacacaaacgtggtacccatttccgcctgttcttcctctttCTTTTGCCATT

Table S1 (related to STAR Methods)

Table S1 (related to STAR Methods)

Selected candidate gene promoter region sequences for Sequoia binding test via ChIP assay. Promotor regions were searched for and selected using the EPD database: https://epd.epfl.ch//index.php
